# Comparison of Zinc Concentrations in the Broth of Commercial Automated Susceptibility Testing Devices (Vitek 2, MicroScan, BD Phoenix, and Sensititre)

**DOI:** 10.1128/spectrum.00052-22

**Published:** 2022-04-04

**Authors:** Tomefa E. Asempa, Christian M. Gill, Vindana Chibabhai, David P. Nicolau

**Affiliations:** a Center for Anti-Infective Research and Development, Hartford Hospital, Hartford, Connecticut, USA; b Department of Clinical Microbiology and Infectious Diseases, School of Pathology, Faculty of Health Sciences, University of the Witwatersrand, Clinical Microbiology Laboratory, Charlotte Maxeke Johannesburg Academic Hospital, National Health Laboratory Service, Johannesburg, South Africa; c Division of Infectious Diseases, Hartford Hospital, Hartford, Connecticut, USA; Instituto Oswaldo Cruz

**Keywords:** antimicrobial resistance, metalloenzymes, susceptibility testing

## Abstract

Up to 4-fold differences in zinc concentrations have been observed in commercial broth routinely utilized for susceptibility testing via manual broth microdilution. Herein, we report the concentration of zinc in the broth of common automated susceptibility testing (AST) platforms (Vitek, MicroScan, BD Phoenix, and Sensititre). For AST platforms with lyophilized broth contents (Vitek and MicroScan), wells were rehydrated with appropriate diluent, and contents were aliquoted out for zinc assay. Aliquots from the manufacturer-specific broth (premade cation-adjusted Mueller-Hinton broth [caMHB]) for BD Phoenix and Sensititre were also assayed by inductively coupled plasma mass spectrometry. Up to a 10-fold difference in zinc concentrations was observed across the 4 platforms (MicroScan: 0.46 mg/L; BD Phoenix: 1.16 mg/L; Vitek: 1.22 mg/L; Sensititre: 4.49 mg/L). Attention should be given to the supraphysiologic and variable zinc concentrations observed in broth used in automated platforms and the subsequent implications for susceptibility testing of metallo-β-lactamase (MBL)-harboring isolates. This variability also hampers efforts to develop a standardized method to uniformly reduce zinc concentrations in broth and mimic physiologic zinc conditions.

**IMPORTANCE** Growing data on the impact of extracellular zinc concentration on metallo-β-lactamase-mediated resistance has shed light on the importance of susceptibility testing media. However, there are no studies documenting the amount of zinc in commonly utilized automated susceptibility testing (AST) platforms. This study reveals supraphysiologic zinc concentrations as well as large zinc variability among AST platforms and highlights the challenges this raises in the development of zinc-limited media.

## OBSERVATION

In a previous study, we observed variability in zinc concentration across commercial brands of cation-adjusted Mueller-Hinton broth (caMHB), resulting in different classifications of meropenem susceptibility among several metallo-β-lactamase (MBL)-harboring *Enterobacterales* isolates (1). We received encouraging feedback from various experts, including a letter from Rennie describing his experience with the manufacturing of caMHB as it pertains to zinc concentrations (1–3). To supplement the work done profiling zinc concentrations in caMHB (the recommended medium for manual broth microdilution trays) (4), this study sought to bridge our knowledge gap of zinc concentrations in the panel/broth of 4 common automated susceptibility testing platforms (Vitek, MicroScan, BD Phoenix, and Sensititre).

Each Vitek 2 Gram-negative susceptibility card (bioMérieux Inc, Durham, NC) has 64 microwells containing dehydrated culture medium with or without an antibiotic (5). Five microwells on one card were each rehydrated with 25 μL of 0.45% sodium chloride (5). The card was placed in an incubator for 2.5 h, after which the contents of each well were aliquoted out and pooled to obtain a total volume of approximately 125 μL. This yielded sufficient volume (>100 μL) to conduct zinc analysis as described below. This was repeated on two additional cards and across two different manufacturer lots.

The 96 wells in the MicroScan Gram-negative panel (Beckman Coulter, Brea, CA) also contain dehydrated culture medium with or without an antibiotic (6). A control (no antibiotic) and antibiotic well (meropenem 1 mg/L) were each rehydrated with 115 μL of distilled water (7). The panel was placed in an incubator for 2.5 h, after which the contents of each well were aliquoted out to obtain individual samples of approximately 115 μL. This was repeated on two additional panels with the same manufacturer lot number.

In contrast to the Vitek and MicroScan panels, the BD Phoenix (Becton, Dickinson and Company, Sparks, MD) (8) and Sensititre (Thermo Fisher, Waltham, MA) (9) panels contain only dehydrated antibiotics and require rehydration with liquid culture medium by the end user using manufacturer-supplied broth. Thus, samples (100 μL) from three manufacturer lots of BD Phoenix automated susceptibility testing (AST) broth (Becton, Dickinson and Company, Sparks, MD) and Sensititre (Thermo Scientific) caMHB with *N*-tris(hydroxymethyl)methyl-2-aminoethanesulfonic acid (TES; Remel Inc. KS, USA) were obtained.

Zinc in each 100-μL broth aliquot from each device was measured by inductively coupled plasma mass spectrometry (ICP-MS). ICP-MS analysis was performed by PureHoney Technologies (Billerica, MA) using an Agilent 7500 CE instrument (see the supplemental material for methods). Intrabatch and interbatch precision (percent coefficient of variation [% CV]) for all standards and samples was ≤10%.

**FIG 1:**
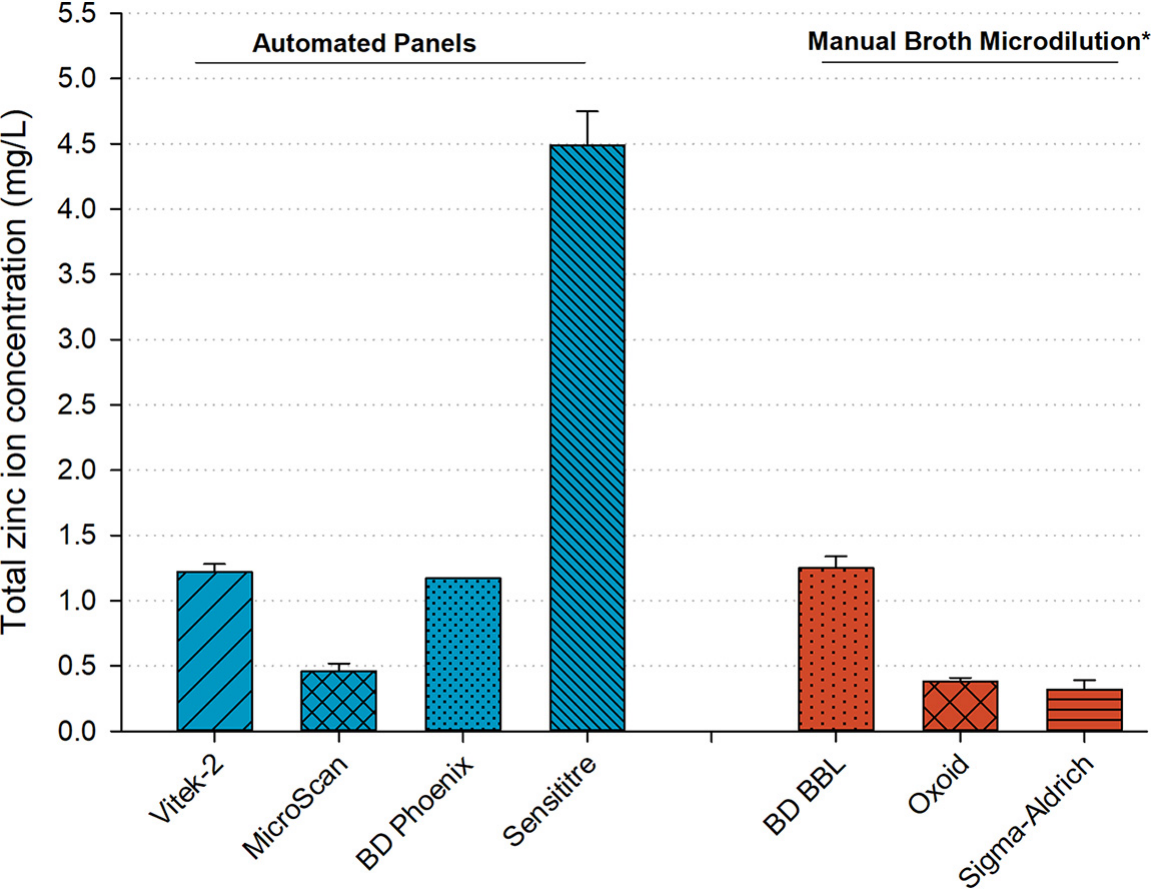
Mean zinc concentrations in the panels or broths of four commercial automated susceptibly testing platforms compared with three commercial dehydrated MHB samples used for manual broth microdilution. The asterisk (*) indicates data obtained from Bilinskaya et al. (1).

Up to a 10-fold difference in mean (standard deviation) zinc concentrations was observed across the four platforms (range of 0.46 [0.06] mg/L [MicroScan] to 4.49 [0.26] mg/L [Sensititre]; Table 1). This difference was larger than the 4-fold difference observed across caMHB commercial brands used for manual broth microdilution trays (range of 0.38 [0.03] mg/L [Sigma-Aldrich] to 1.25 [0.09] mg/L [BD BBL]; Fig. 1) (1). With each platform, well-to-well and lot-to-lot variability was minimal and was concordant with FDA package labeling that describes precise distribution of lyophilized broth across the wells with only the antibiotic concentration varying. The zinc concentration (component of broth) in each reconstituted well is therefore not expected to vary across wells on a single tray, providing rationale for this study’s sampling scheme.

**TABLE 1:** Total zinc concentrations in the panels or broths of four commercial automated susceptibly testing platforms

		Zinc concn (mg/L)		
Product	Lot no.	Replicate 1	Replicate 2	Replicate 3
Vitek 2 Gram-negative susceptibility card AST-N255 (reference no. 413724)	6551674503	1.27	1.24	1.22
	6551578403	1.14	1.17	1.29
Microscan Gram-negative panel MIC 44 (catalog no. B1016-175)	2021-04-17 (control well)	0.57	0.49	0.46
	2021-04-17 (meropenem well)	0.39	0.42	0.44
BD Phoenix AST broth (catalog no. 246003)	1124221	1.16	1.16	1.17
Sensititre, Thermo Scientific caMHB with TES (product no. T3462)	190583	4.78	4.26	4.44

Profiling the concentrations of zinc in the variety of culture media used in manual and automated susceptibility testing is important because efforts are underway to develop zinc-limited medium through zinc sequestration (via Chelex) or through the addition of EDTA (1, 10). Unfortunately, the effectiveness of these methods depends on the baseline zinc concentration in the medium; thus, zinc variability across broths/panels poses a huge challenge in standardizing a process that can consistently attain an appropriate zinc concentration that mimics physiologic conditions. Notably, the total zinc concentration in human serum ranges from 0.6 to 1.4 mg/L and is highly protein bound (80 to 99%) to albumin and alpha-2-macroglobulin, effectively reducing the amount of zinc freely available to interact with cells (1, 11).

It is also important to note that the variability in zinc concentration observed in this current study (range of 0.46 mg/L to 4.49 mg/L) is not expected to result in clinically meaningful *in vitro* changes in already elevated carbapenem MICs among MBL-harboring isolates, as even the lowest of these “supraphysiologic” zinc concentrations (i.e., 0.46 mg/L) is multiple-fold higher than physiologic free zinc concentrations, and, thus, zinc is still readily available for use by metallo-β-lactamases to hydrolyze meropenem (continuing to result in elevated MICs) (1, 12–14). This was demonstrated in a previous study evaluating commercial broths for manual broth microdilution (1). Therein, caMHB from three different manufacturers had such high concentrations of zinc (0.3 mg/L to 1.3 mg/L) that we did not observe significant differences in meropenem MICs (i.e., the majority of isolates were meropenem MIC of ≥32 mg/L) until a specific lot of broth (Sigma-Aldrich; product number 90922, lot number BCCB1508) was identified that had zinc in the range of 0.2 to 0.3 mg/L, resulting in variable and susceptible meropenem MICs among several MBL-harboring isolates (1). Further proof-of-concept studies utilizing a range of EDTA concentrations to gradually reduce zinc concentrations in broth resulted in clinically meaningful reductions in meropenem MICs, demonstrating the contribution of zinc (1). Certainly, further *in vitro* and *in vivo* studies are warranted to inform clinical relevance.

A similar trend between calcium and susceptibility results was documented for daptomycin several decades ago (15, 16). Relative to the physiologically relevant Ca^2+^ concentration of 50 mg/L that is recommended for addition to broth for susceptibility testing (17), a reduction to 25 mg/L resulted in a 2- to 4-fold decrease in activity, while at concentrations above 50 mg/L (i.e., 75 mg/L), susceptibility rates were similar to results obtained with 50 mg/L (15).

In summary, a 10-fold difference in zinc concentrations was observed across the media utilized in four commonly used automated susceptibility testing platforms. In support of efforts to develop AST media that better reflect physiologic conditions, documenting the baseline concentrations of zinc, an essential cofactor of MBL, is an essential first step in optimizing the clinical predictive value of AST and the screening of therapies for the treatment of infections caused by MBL-harboring isolates.
