# Supplementary material for: Comparison of Zinc Concentrations in the Broth of Commercial Automated Susceptibility Testing Devices (Vitek 2, MicroScan, BD Phoenix, and Sensititre)
Source: Microbiol Spectr. 2022 Apr 4;10(2):e00052-22. doi: 10.1128/spectrum.00052-22 (PMC9045177; doi:10.1128/spectrum.00052-22)
Supplement: SUPPLEMENTAL FILE 1 — Supplemental material. Download SPECTRUM00052-22_Supp_1_seq5.pdf, PDF file, 0 MB [file spectrum00052-22_supp_1_seq5.pdf]

Zinc analysis was performed by ICP-MS with Cobalt used as an internal standard. Briefly a 250 ppb Cobalt solution (Agilent catalog# 5190-8346) 2% Nitric acid solution (trace metal grade) was prepared as the sample diluent. A 12-point serial dilution of zinc standard (Supelco HCO1344606) starting at 2,500 ppb was prepared in the 2% Nitric Acid with 250 ppb Cobalt IS. Bacterial broth samples (100µL to 400µL in volume) were diluted with the addition of 3.0 mL of the 2% Nitric Acid solution with Cobalt IS and allowed to sit at room temperature for 1 hour. All samples and standards were prepared in 12 mm polyethylene test tubes. The ICP-MS analysis was performed on an Agilent 7500ce ICP-MS instrument with an Argon plasma. Cobalt IS was monitored at 59 g/mol and the 64 g/mol isotope of Zinc was used for the analysis. The Zinc signal in the standards and samples was normalized to the Cobalt IS and the concentration determined using a standard curve with a linear regression curve fit (Microsoft Excel).
